# Mechanical Characteristics of Individualized Biodegradable Augmentation Scaffold—In Vitro Pilot Study

**DOI:** 10.3390/ma17061419

**Published:** 2024-03-20

**Authors:** Roko Bjelica, Vladimir Prpić, Nenad Drvar, Amir Ćatić, Dragana Gabrić

**Affiliations:** 1 Department of Oral Surgery, School of Dental Medicine, University of Zagreb, 10000 Zagreb, Croatia; rbjelica@sfzg.hr; 2 Department of Fixed Prosthodontics, School of Dental Medicine, University of Zagreb, 10000 Zagreb, Croatia; vprpic@sfzg.hr (V.P.); catic@sfzg.hr (A.Ć.); 3 Topomatika, LLC, 10431 Sveta Nedelja, Croatia; n.drvar@topomatika.hr; 4 University Dental Clinic, University Hospital Centre Zagreb, 10000 Zagreb, Croatia

**Keywords:** bone regeneration, alveolar ridge augmentation, polymers, printing, three dimensional

## Abstract

The alveolar ridge reconstruction of vertical and combined bone defects is a non-predictable procedure with varying percentages of success. The greatest challenge for vertical and combined bone augmentation is to maintain mechanical stability of the bone graft; therefore, it is mandatory to provide and preserve space for bone regeneration. The development of biomaterials and 3D printing has enabled the use of polymer scaffolds in the reconstruction of alveolar ridge defects. The aim of this pilot study was to evaluate the mechanical characteristics of an innovative individualized biodegradable polylactic acid (PLA) scaffold, under dynamic conditions, simulating biodegradation and the influence of masticatory forces. After the design and 3D printing of PLA scaffolds, two groups of 27 scaffolds were formed according to the compression testing procedure. The compression tests were performed in occlusal and lateral directions. In each of the two groups, nine subgroups of three scaffolds were formed for different testing periods during in vitro degradation with a total period of 16 weeks. Results showed that biodegradation and load application had no significant influence on mechanical characteristics of tested scaffolds. It can be concluded that simulated masticatory forces and biodegradation do not significantly influence the mechanical characteristics of an individualized biodegradable augmentation scaffold.

## 1. Introduction

Bone augmentation is an oral surgical procedure that replaces the insufficient volume of the edentulous alveolar ridge and is most commonly used to enable the placement of dental implants [1]. Adequate ridge dimensions are essential for providing sufficient aesthetic and functional results. Following tooth extraction, alveolar ridge resorption is a common and unavoidable process, which results in significant dimensional changes [2]. The reduction that can be expected after multiple tooth extractions is up to 50% of the original ridge width, with greater bone resorption in the distal region [3]. Allen et al. [4] classified alveolar ridge defects in three different classes regarding the resorption axis (class A or vertical defects, class B or horizontal defects, and combined defects as class C) and introduced the concept of severity, assessing defects of less than 3 mm as mild, 3 to 6 mm as moderate, and those greater than 6 mm as severe. The severity of the defect influences the success of augmentation techniques and poses a critical issue for successful dental implant placement [5]. Augmentation techniques of horizontal defects give predictable results in contemporary oral surgery [5], while results in vertical and combined defects are less predictable with a lower percentage of success [6]. The four key factors for successful alveolar ridge augmentation are the space maintenance, stability of the fibrin clot, exclusion of the epithelium and connective tissue, and primary wound closure [7].

Considering these key principles and the fact that the greatest challenge for vertical and combined bone augmentation is to maintain mechanical stability of the bone graft, it is crucial to know the membranes and meshes frequently used in modern guided bone regeneration (GBR). Apart from preventing undesired soft tissue ingrowth, barrier membranes have a significant role in space maintenance and ensuring mechanical stability of the bone graft [8]. Consequently, the well-known resorbable collagen membranes are not the material of choice when vertical bone gain is required, regardless of their proven effectiveness in horizontal ridge augmentation [9]. Different therapeutic approaches have been proposed for combined and vertical ridge augmentation, of which the most common are using polytetrafluoroethylene (PTFE) membranes, titanium meshes, and autogenous bone blocks [10]. The main advantages of titanium meshes and PTFE membranes are that they are able to maintain the space without collapsing, with simultaneous ability of adaptation and shaping during the surgery [11]. However, there are certain complications of using PTFE membranes and titanium meshes, such as wound dehiscence and subsequent bacterial colonization. Another major disadvantage is the need for a second surgery of membrane or titanium mesh removal [1]. The autogenous bone block technique, described by Khoury et al. [12], is also associated with drawbacks and complications, primarily the infection of the bone graft and disturbances observed in donor sites [13]. Although clinical and experimental studies of aforementioned ridge augmentation techniques have shown acceptable treatment results, they also demand exceptionally skilled surgeons [14].

The development of biomaterials has enabled the use of natural and synthetic polymers in biomedicine with great potential in bone regeneration [15]. Three-dimensional matrices, also known as scaffolds, play a vital role in bone regeneration by providing a stable space for bone cell adhesion, proliferation, and differentiation [16]. An ideal polymeric scaffold should be non-cytotoxic, biodegradable, printable, bioactive, and osteoconductive in vivo [17]. Synthetic polymers are considered more suitable for biomedical use due to the possibility of exact control of the design, degradation rate, and modification of biochemical, physical, and mechanical characteristics [15]. For these reasons, polylactic acid (PLA) is a good candidate polymer scaffold in bone tissue engineering [18]. It is a biodegradable semi-crystalline biomaterial derived from 100% renewable resources with versatile properties [19]. Even though the applications of PLA as a bone regeneration material were originally orthopedic [20], it has found its role in craniofacial and alveolar bone regeneration [21]. It undergoes hydrolytic degradation into lactic acid, which is naturally present in the body [22]. One of the oldest 3D additive manufacturing technologies, fused deposition modeling (FDM), enables the production of a PLA scaffold in a relatively fast, inexpensive, and well-explored way [23]. Due to its minimal thermal expansion coefficient, PLA demonstrates resistance to warping, enhancing its dimensional accuracy, and consequently positioning it as a suitable material for FDM [24]. The ongoing development of Computer-Aided Design (CAD) technology and valuable diagnostic tools, such as cone beam computer tomography (CBCT) and intraoral scanning, encourages researchers to implement the use of individually designed and fabricated scaffolds in daily practice [25].

The use of the PLA scaffold in the reconstruction of alveolar ridge defects has shown promising results, but currently the number of studies and clinical cases remains scarce [26,27,28]. Despite its great potential in bone augmentation procedures, there is still limited evidence to draw conclusions or give guidelines usable in everyday clinical practice [29]. There is also a lack of in vitro studies defining the mechanical properties of such scaffolds under dynamic conditions. The Finite Element Method (FEM) is widely employed in the research of polymer materials for its ability to simulate and analyze complex structural and mechanical behaviors [30]. In the context of polymer research, FEM is utilized to model and predict the response of materials to various loading conditions. This study addressed the limitations of current techniques by exploring a novel approach utilizing a 3D polymeric scaffold, specifically PLA. By focusing on the mechanical aspects of polymeric scaffolds, this research has been focused on providing valuable insights into their potential for alveolar ridge reconstruction. The integration of a FEM analysis in the research served as a tool for predicting and understanding the mechanical behavior of the PLA scaffold under diverse loading scenarios before experimental validation with mechanical tests.

Taking into consideration these findings, the aim of this study was to evaluate the mechanical characteristics of an individualized biodegradable augmentation scaffold, under dynamic conditions, simulating biodegradation and the influence of masticatory forces.

## 2. Materials and Methods

### 2.1. The Design and 3D Printing of PLA Scaffolds

This in vitro pilot study was conducted according to the guidelines of the Declaration of Helsinki and was approved by the Ethics Committee of the School of Dental Medicine, University of Zagreb (protocol code: 05-PA-30-19-6/2023 on 15 June 2023). Individualized biodegradable augmentation scaffolds were designed and fabricated at Topomatika, LLC, Sveta Nedelja, Croatia. Mechanical characteristics of the scaffolds were tested at the experimental laboratory of the School of Dental Medicine, University of Zagreb.

Synthetic PLA polymer Lactoprene^®^ 7415 (Poly-Med Inc., Anderson, SC, USA) was a material of choice for the fabrication of scaffolds used in this study. It is registered with the Food and Drug Administration (FDA) as a medical device and has been registered by the International Organization for Standardization (ISO) 13485 standard [31].

**Figure 1:**
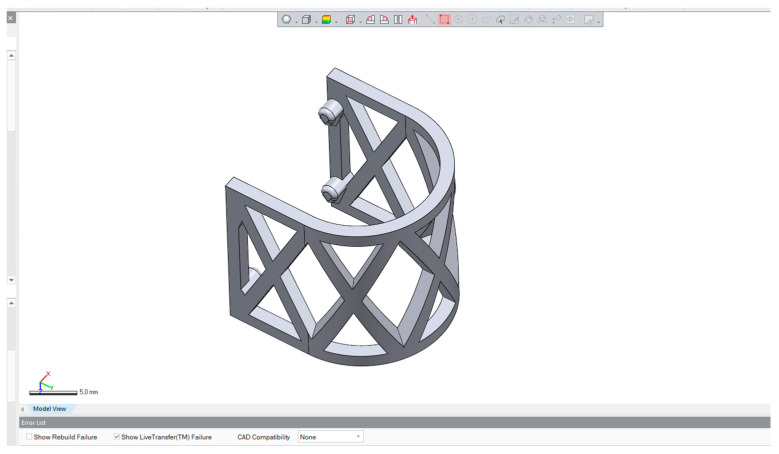
The design of the semicylindrical scaffold in Geomagic Design X Version 2020.0.1CAD software.

**Figure 2:**
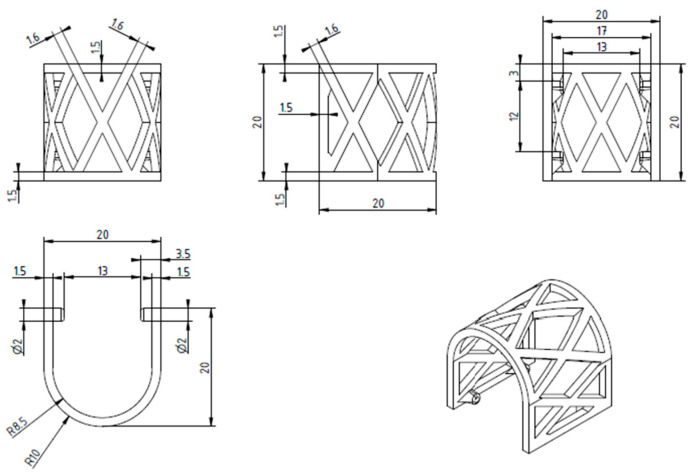
The geometry of the PLA scaffold.

**Figure 3:**
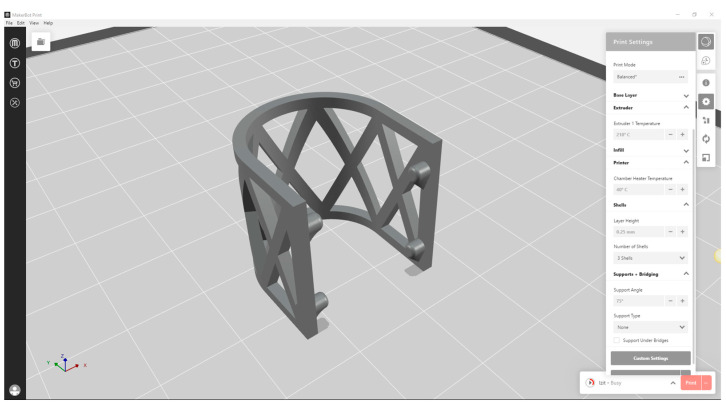
CAD of the scaffold with 3D printing parameters imported to MakerBot Print 4.10.1.

The scaffolds were designed in CAD software Geomagic Design X Version 2020.0.1 (Artec 3D, Senningerberg, Luxembourg) (Figure 1). The shape of the scaffold was semicylindrical, allowing the repeatable mechanical testing procedure (Figure 2). The scaffolds were 3D-printed using a MakerBot Method X Carbon fiber (MakerBot Industries, New York, NY, USA) printer, equipped with a Labs Gen 2 experimental extruder. MakerBot Print 4.10.1 (MakerBot Industries, New York, NY, USA) was used to import and prepare CAD models for printing (Figure 3). The printing parameters were set at a 210 °C extruder temperature, 40 °C chamber temperature, and layer height of 0.25 mm. The number of shells was 2, infill was solid with a thickness of 0.8 mm for one shell, and printing speed was set at 50 mm/s.

### 2.2. Finite Element Method (FEM) Analysis of PLA Scaffold

The FEM model was proposed to simulate the structural behavior of the PLA scaffold prior to the mechanical tests, enabling a realistic simulation of the scaffold’s response under different loading conditions simulating masticatory forces. The purpose of the FEM analysis was to determine whether the scaffold could withstand the required forces without breaking and/or deformation exceeding 4 mm.

#### 2.2.1. Scaffold Geometry and Meshing

The geometry of the analyzed scaffold was defined in CAD software mentioned in the previous section (Geomagic Design X Version 2020.0.1) and imported to the commercial FEM software Ansys Mechanical 2022 R1 (Ansys Inc., Canonsburg, PA, USA). The finite element mesh was generated to discretize the geometry into smaller elements, ensuring an accurate representation of the structural features. The meshing process employed triangular elements.

#### 2.2.2. Physical and Mechanical Properties of Lactoprene^®^ 7415

Material properties for the inspected material are shown in Table 1.

#### 2.2.3. Loading Conditions

**Figure 4:**
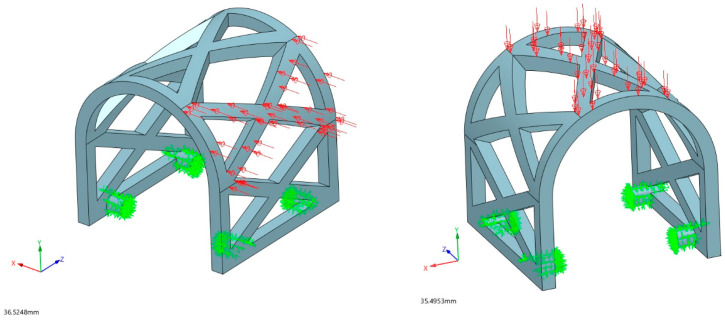
Lateral (**left**) and occlusal (**right**) loading scenarios on PLA scaffold sample (red arrows). The attachments for PLA scaffold fixation in vivo were solidified for FEM purposes (green arrows).

Two different loading scenarios were used for the simulation of occlusal and lateral masticatory forces and their effects on the stress and deformation of the PLA scaffold (Figure 4). Considering the mean values of the masticatory forces in the literature [32,33], the values of 150 N for occlusal load and 50 N for lateral load were determined. Occlusal load refers to the vertical force exerted on the scaffold during the process of biting and chewing in vivo, while the lateral load involves horizontal forces induced by the tongue and surrounding muscles.

### 2.3. Compression Testing Procedure

**Figure 5:**
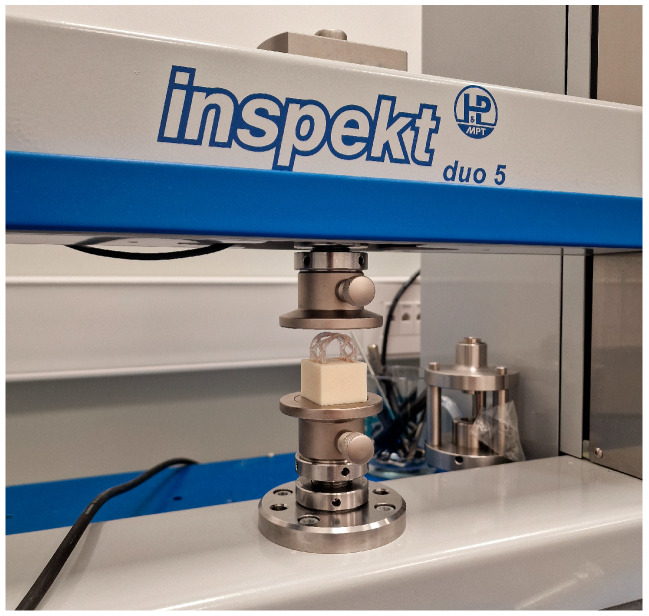
Occlusal compression test on Universal testing machine Inspekt table 50 kN.

**Figure 6:**
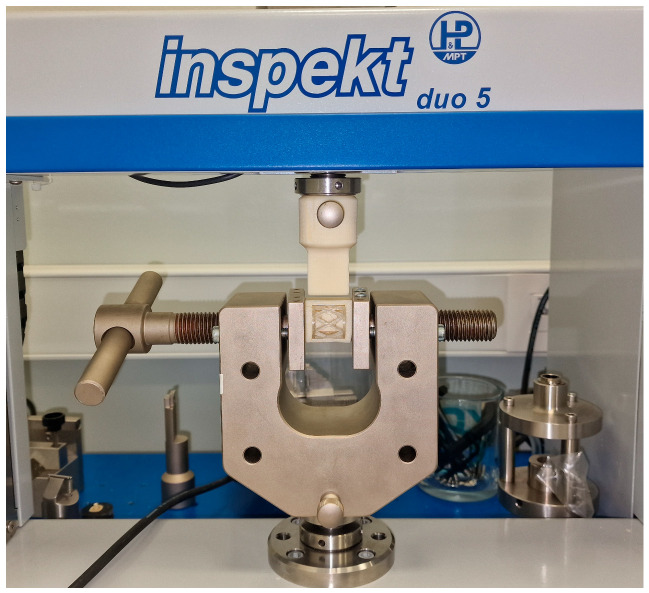
Lateral compression test on Universal testing machine Inspekt table 50 kN.

Mechanical characteristics of 3D-printed scaffolds were evaluated under dynamic in vitro conditions for a total period of 16 weeks. Universal testing machine Inspekt table 50 kN (Hegewald & Peschke Meß—und Prüftechnik GmbH, Nossen, Germany) was used for compression tests. The tests were performed in two directions simulating masticatory forces:

1. Occlusal compression test (150 N) (Figure 5).
2. Lateral compression test (50 N) (Figure 6).

The compression tests were conducted at a 0.5 mm/min speed. Break criteria for the occlusal compression test were set to the following parameters: a 150 N load, 4 mm stroke, and 80% load decrease. The lateral compression test had the same break criteria except the load, which was set at 50 N.

Force–displacement graphs were created with LabMaster 31.8 software (Hegewald & Peschke Meß—und Prüftechnik GmbH, Nossen, Germany).

The total of 54 scaffolds were 3D-printed and divided into two main groups of 27 scaffolds according to the aforementioned mechanical testing procedure. The number of 27 scaffolds for each group was obtained by the sum of nine different test periods of 3 scaffolds. The sample size of three scaffolds per subgroup was determined according to the recent in vitro study of novel bone scaffolds based on 3D-printed PLA and cell-laden alginate hydrogel by Noroozi et al. [34].

1. Group 1 comprised 27 Lactoprene^®^ 7415 scaffolds for occlusal compression testing.
2. Group 2 comprised 27 Lactoprene^®^ 7415 scaffolds for lateral compression testing.

In each of the main groups (1 and 2), nine subgroups of three scaffolds were formed for different testing periods as follows: an initial (dry) test, 2-week test, 4-week test, 6-week test, 8-week test, 10-week test, 12-week test, 14-week test, and 16-week test.

### 2.4. Biodegradation of the Scaffolds

**Figure 7:**
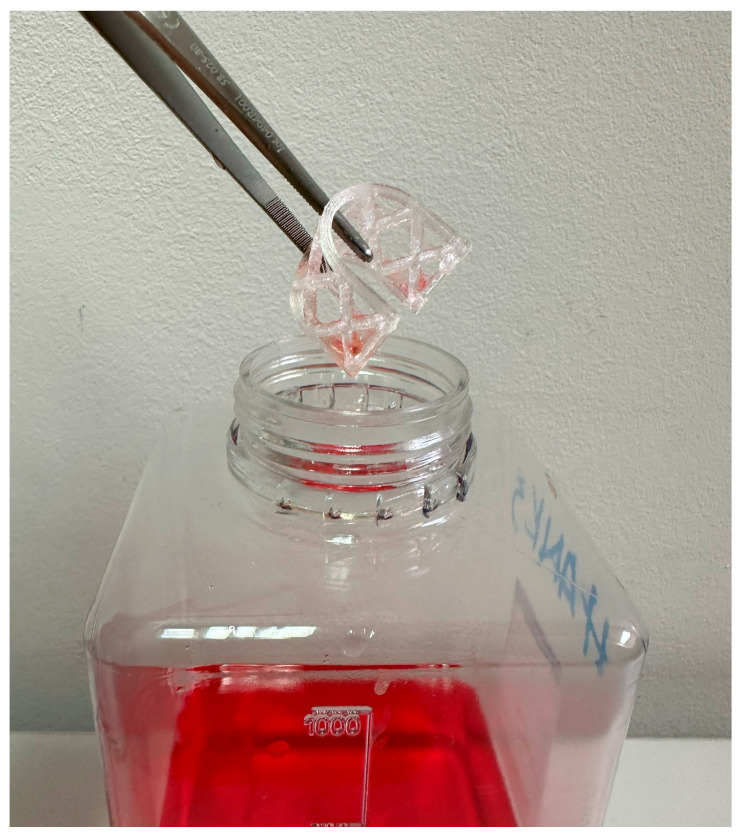
Biodegradable augmentation scaffold removed from Hank’s balanced salt solution using sterile stainless steel forceps.

Following the compression testing procedure of the initial subgroup of dry scaffolds, the rest were soaked in sample bottles NK100GT behrotest (behr Labor-Technik GmbH, Düsseldorf, Germany) filled with Hank’s balanced salt solution (HBBS) (Biowest, Nuaille, France) for 16 weeks and a temperature of 22 °C. HBBS served as a dissolving agent simulating biodegradation. Stainless steel forceps (Henry Schein, Melville, NY, USA) were used to remove scaffold subgroups from sample bottles every 2 weeks for both occlusal and lateral compression tests (Figure 7). The scaffolds were dried on sterile gauze (Paul Hartmann AG, Heidenheim, Deutschland) for 2 min before the mechanical testing procedure.

### 2.5. Compressive Modulus of the Scaffold

The compressive modulus, E_c_, of the scaffolds was calculated according to ISO 604 [35]. The force–displacement graphs were used to determine the ratio of the force difference to the corresponding displacement difference.

Due to the geometry of the individualized biodegradable augmentation scaffold and its mesh-like structure, the exact cross-section of the specimen was not obtainable. In this regard, the protocol for the calculation of the compressive modulus was based on the protocol already used for the compressive modulus in the literature [34,36].

Considering the slower biodegradation of Lactoprene^®^ [37] compared to the longest immersion period of 16 weeks, the cross-section value was constant for every scaffold used in the presented study. Thus, the measured force difference with a suitable measurement unit was used as equivalent stress.

Universal testing machine Inspekt table 50 kN was equipped with a deformation indicator suitable for determining the change in length of the appropriate part of the test specimen; thus, compressive strain was calculated according to ISO 604.

According to these values, the calculated compressive modulus should be considered as the equivalent compressive modulus.

### 2.6. Statistical Analysis

Due to the small sample size (n = 3 per experimental subgroup), a non-parametric approach (Kruskal—Wallis test) was used to compare the values of the compressive modulus among time points. As the omnibus test yielded a statistically non-significant result, no post–hoc comparisons were performed. The statistical analysis was performed using SPSS (version 25; IBM, Armonk, NY, USA) with a significance level of 0.05.

## 3. Results and Discussion

### 3.1. FEM Analysis

#### 3.1.1. Occlusal Loading

**Figure 8:**
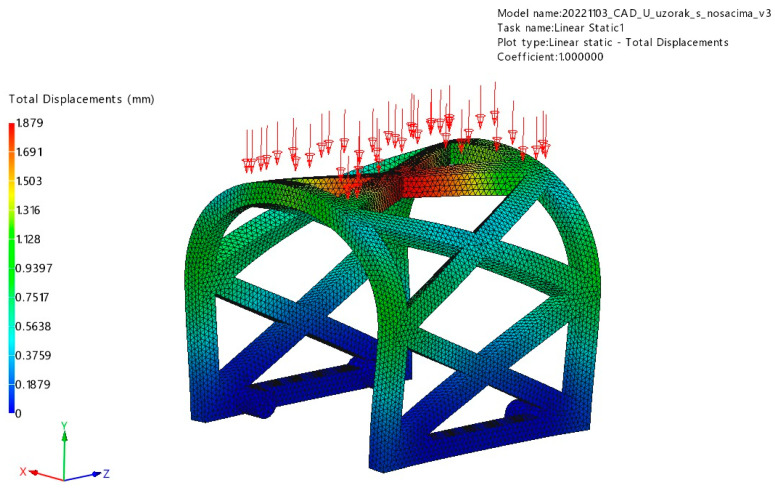
Displacement results of the occlusal loading (red arrows) FEM analysis.

**Figure 9:**
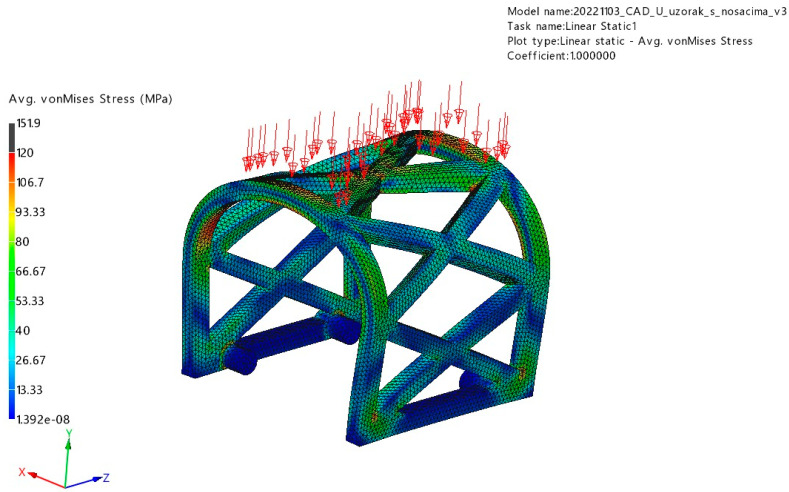
Von Mises stress results of the occlusal loading (red arrows) FEM analysis.

The stress and displacement results for occlusal loading are presented in Figure 8 and Figure 9. Based on this analysis, the PLA scaffold was able to withstand 150 N of occlusal force without breaking or deformation exceeding 4 mm (maximum displacement was 1.88 mm). The maximum stress obtained in the simulation was 151.9 MPa.

#### 3.1.2. Lateral Loading

**Figure 10:**
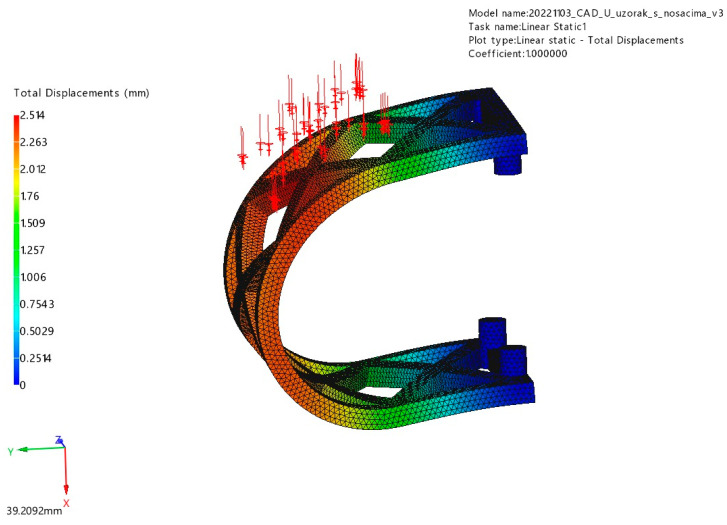
Displacement results of lateral loading (red arrows) FEM analysis.

**Figure 11:**
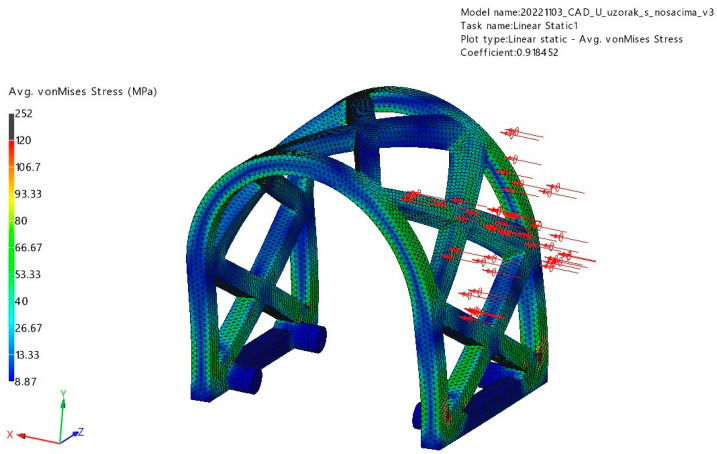
Von Mises stress results of lateral loading (red arrows) FEM analysis.

The results for stress and displacement of the laterally loaded PLA scaffold are presented in Figure 10 and Figure 11. This sample also withstood a determined force of 50 N uneventfully with maximum displacement of 2.51 mm. The maximum stress in this simulation was 252 MPa.

### 3.2. Occlusal Compression Test

**Figure 12:**
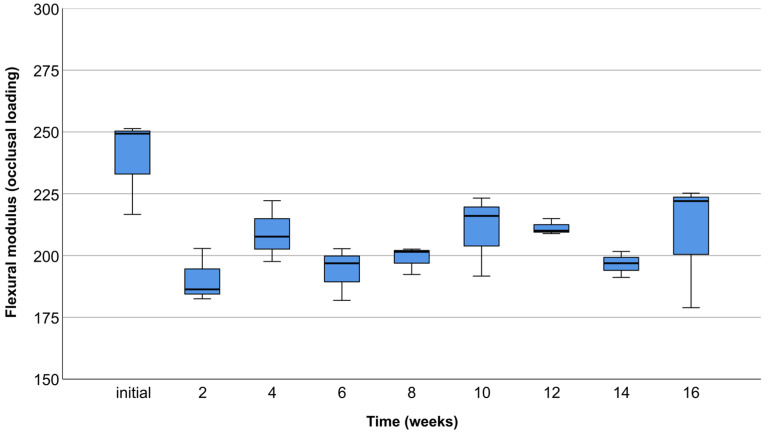
Box plot chart showing compressive modulus of occlusal compression test scaffolds in MPa for different testing periods.

The compressive modulus measured with occlusal compression tests showed no statistically significant difference among time points (*p* = 0.119) (Figure 12).

### 3.3. Lateral Compression Test

**Figure 13:**
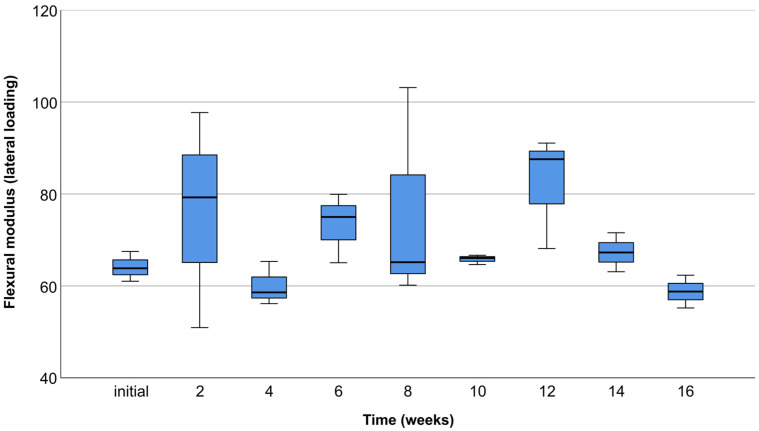
Box plot chart showing compressive modulus of lateral compression test scaffolds in MPa for different testing periods.

The compressive modulus measured with lateral compression tests showed no statistically significant difference among time points (*p* = 0.175) (Figure 13).

In the presented study, the mechanical characteristics of the individualized biodegradable augmentation PLA scaffold were evaluated under dynamic conditions. The FEM analysis played a crucial role in comprehensively assessing the mechanical properties of the scaffold sample. Prior to conducting compression tests and simulating biodegradation, FEM served as a computational tool to predict and analyze the scaffold’s behavior under different loading conditions. This approach allowed for a virtual exploration of the scaffold’s response to simulated masticatory forces, providing insights into its mechanical characteristics before experimental validation. Subsequently, the mechanical tests and biodegradation simulations were conducted, revealing that the simulated masticatory forces and biodegradation have not significantly influenced the mechanical characteristics of the individualized biodegradable augmentation scaffold.

According to Parra et al. [29], the variability of clinical outcomes and limitations of the reviewed studies do not permit a meta-analysis of results. Consequently, there is a limit in defining the best application of PLA. PLA was initially used as a resorbable membrane in alveolar ridge augmentation [38]. It was also used by Serino et al. [39] for the preservation of post-extraction alveoli with significantly lower bone resorption compared to the control group. The introduction of cone beam computer tomography (CBCT) and Computer-Aided Design/Computer-Aided Manufacturing (CAD/CAM) technology enabled the fabrication of individualized polymer scaffolds suitable for the augmentation of large alveolar ridge defects [40]. It is worth mentioning that scaffolds used in the augmentation of large bone defects need to be fixated with screws to maintain stability. It is precisely why PLA is a valuable material, with another application in manufacturing of fixation screws [41]. The complications associated with biodegradable PLA screws are relatively low, along with good mechanical characteristics, similar to those obtained by widely used titanium screws [42]. Matsuo et al. [26] were the first authors who reported the use of custom-made biodegradable PLA mesh reinforced with hydroxyapatite (HA) in the reconstruction of large defects of the mandible. The postoperative course was uneventful, and the bone quality of the reconstructed mandibular area was measured with obtained values comparable to the native bone. In a recent study by Suzuki et al. [40], it was demonstrated that a PLA-based nanoscale architecture scaffold has the appropriate degree of mechanical strength, providing a suitable microenvironment for bone formation. The mechanical characteristics of augmentation scaffolds influence the cell differentiation ability; thus, a sufficient level of mechanical strength is required for successful bone regeneration therapy [17]. The maximum compressive modulus values exceeding 250 MPa in occlusal compression tests, and 90 MPa in lateral compression tests, indicate the desired stiffness for optimal bone regeneration outcomes. From a guided bone regeneration point of view, scaffolds with high stiffness favor the osteogenic differentiation of cells [43]. Taking into consideration the importance of appropriate mechanical properties in biomedical applications, it was essential to perform multidirectional compression tests with sufficient load closely resembling in vivo conditions. The specified loads of 50 N for lateral and 150 N for occlusal tests were carefully determined considering the mean masticatory forces in the literature [32,33]. The FEM analysis emerged as an indispensable preliminary assessment tool, offering valuable insights into the scaffold’s mechanical characteristics under different loading conditions. The decision to proceed with multidirectional compression tests was contingent upon the assurance provided by FEM results, confirming the scaffold’s ability to withstand predetermined forces. In essence, FEM played a pivotal role in guiding and validating subsequent experimental investigations, ensuring a comprehensive understanding of the scaffold’s mechanical performance. Abe et al. [44] observed that a poly (lactic acid/caprolactone) (PLCL) bilayer membrane possesses suitable mechanical strength combined with high breaking strain compared to the control group. In vitro degradation did not influence the mechanical characteristics, which is in accordance with the results obtained in the presented study. However, it is necessary to mention that the addition of the polycaprolactone (PCL) decreased the degradation rate. Furthermore, Thomas et al. [45] found that an HA-incorporated 3D-printed PLA scaffold showcased compressive strength (13.4 ± 5.53 MPa) adequate for future in vivo evaluation. In this regard, synthetic PLA polymer Lactoprene^®^ 7415 was a material of choice in the presented study, due to its strictly determined mechanical properties and degradation rate. The key factor is establishing a balance between the mechanical resistance and biodegradation process [29]. Lactoprene^®^ 7415 exhibits a controlled strength decline in about 3 to 9 months and mass loss in about 12 to 36 months, depending on the processing, physiological environment, and anatomical location [37]. In the presented study, it was necessary to maintain the original form and mechanical characteristics of scaffolds within the specified period of in vitro degradation. The 16-week period aligns with the critical phases of bone healing and regeneration, capturing the transition from early bone formation to a more mature and stable state [46]. Therefore, Lactoprene^®^ 7415 as the material for scaffold samples aligns logically with the specified 16-week in vitro degradation period. This material’s controlled degradation aligns with the study’s objective to assess scaffold performance within a relatively short duration, ensuring meaningful insights into its mechanical behavior and potential applications in alveolar ridge reconstruction. According to the recent in vitro study by Noroozi et al. [34], the nominal stress—strain and force—displacement curves are associated with errors. These authors claim that force–displacement graphs do not provide the readers with a deep understanding of the mechanical characteristics of the fabricated scaffolds. In addition, they found that calculating equivalent values based on nominal and real geometries of the scaffolds gives differing results. Nevertheless, since the geometry of 3D-printed polymer scaffolds used for alveolar ridge reconstruction is complex and varies depending on the clinical case, it is common for researchers to use nominal and equivalent values in the calculation of the elastic modulus [34]. Moreover, the constitutive behavior of scaffold material is nonlinear, especially in the case of polymers, which requires more parameters for description [36]. Therefore, the simple approach is accepted, where the stress–strain relation of a scaffold in the range of reversible deformation is interpreted as the elastic modulus [36].

In the context of the presented study, the decision not to directly compare FEM analysis results (Von Mises stress and displacement of the scaffold) with compression tests stems from the aforementioned interpretation of mechanical characteristics, particularly the elastic modulus. The rationale for avoiding a direct comparison lies in the understanding that the stress–strain relationship of the PLA scaffold in the reversible deformation range, interpreted as the elastic modulus, is inherently distinct from the Von Mises stress derived through the FEM analysis. The stress–strain relation of the scaffold, especially when dealing with complex and anisotropic geometries inherent in 3D-printed scaffolds used in alveolar ridge reconstruction, is subject to nonlinearities. The constitutive behavior of polymer materials further complicates matters, necessitating a more comprehensive parameterization for an accurate description. Attempting a direct comparison with FEM-derived Von Mises stress would overlook the inherent differences in the underlying interpretations of mechanical characteristics, potentially leading to inaccurate assessments. Additionally, the comparison between these values would be valid only for the first (dry) subgroup of PLA scaffolds due to the biodegradation and consequential volume loss in further subgroups. Thus, in the presented study, it is chosen to rely on comprehensive understanding of the biodegradable augmentation scaffold’s mechanical behavior through a separate analysis, acknowledging the limitation and nuances associated with each method.

In summary, this study does not lack certain limitations. Given the scarce number of in vitro studies investigating the influence of biodegradation and applied load on mechanical characteristics of PLA scaffolds used in bone regeneration, valuable results were attained. It is of utmost importance to re-emphasize that the presented study was an in vitro pilot study, so the sample size of 54 scaffolds was sufficient to analyze their mechanical behavior during the specified biodegradation period. However, considering the large number of different testing periods, a larger sample size would be desirable. Finally, further in vivo research is necessary to fully develop an individualized biodegradable augmentation scaffold with the ability to be used in clinical practice.

## 4. Conclusions

According to the presented results, the biodegradation and application of occlusal and lateral load had no significant influence on mechanical characteristics of the individualized biodegradable augmentation scaffold. Considering the discussed limitations of this study, the PLA augmentation scaffold represents a viable solution from a mechanical point of view. Despite these results, there are still factors that need to be overcome in order to establish more predictable and reliable clinical protocols. These findings give important evidence to support further studies with the intention of creating a standardized protocol for clinical use.

## Figures and Tables

**Table 1:** Physical and mechanical properties of Lactoprene^®^ 7415.

Material Property	Value	Unit
Elastic modulus	3149	MPa
Poisson’s ratio	0.36	
Shear modulus	1157	MPa
Mass density	1240	Kgm^−3^
Tensile strength	40	MPa
Compressive strength	65	MPa
Yield strength	77	MPa
Thermal expansion coefficient	0.000041	K^−1^
Thermal conductivity	0.183	Wm^−1^K^−1^
Specific heat	1300	Jkg^−1^K^−1^
Material damping ratio	2.2	

## Data Availability

Data are contained within the article.
